# Exploring the Potential of the PerioAI System to Support Periodontal Clinical Decision Making: A Proof‐of‐Principle Study

**DOI:** 10.1111/jcpe.70138

**Published:** 2026-05-03

**Authors:** Hairui Li, Yuan Li, Minhui Tan, Zhiming Cui, Dinggang Shen, Andrea Roccuzzo, Maurizio S. Tonetti

**Affiliations:** ^1^ Shanghai Perio‐Implant Innovation Center and Oral Biomedical Intelligence Technology Laboratory (ORAL‐BIT Lab) Institute of Integrated Oral, Craniofacial and Sensory Research, Shanghai Ninth People's Hospital, Shanghai Jiao Tong University School of Medicine Shanghai China; ^2^ College of Stomatology, Shanghai Jiao Tong University National Center of Stomatology, National Clinical Research Center for Oral Diseases, Shanghai Key Laboratory of Stomatology Shanghai China; ^3^ School of Biomedical Engineering & State Key Laboratory of Advanced Medical Materials and Devices ShanghaiTech University Shanghai China; ^4^ Shanghai United Imaging Intelligence Co., Ltd. Shanghai China; ^5^ Shanghai Clinical Research and Trial Center Shanghai China; ^6^ Department of Periodontology, School of Dental Medicine University of Bern Bern Switzerland; ^7^ European Research Group on Periodontology Genova Italy

**Keywords:** artificial intelligence, clinical trial, periodontal prognosis, periodontal treatment plan

## Abstract

**Aim:**

To explore the potential of PerioAI, an artificial intelligence system integrating intraoral scanning and cone‐beam CT, to automatically measure gingival margin‐to‐bone distance (GBD) and convert it into AI‐derived probing depth (AI‐PD), and to evaluate whether AI‐PD may provide additional information to support periodontal clinical decision making when radiographic imaging represents the primary source of available periodontal information.

**Materials and Methods:**

This cross‐sectional proof‐of‐principle study included 53 patients with periodontitis (1298 teeth, 7788 sites). GBD measurements were converted to AI‐PD using validated formulas. Clinical decision making (prognosis and treatment planning) was evaluated by one periodontist under three information conditions: (i) orthopantomogram (OPG) + original periodontal chart (used to establish the reference clinical decision); (ii) OPG‐only; and (iii) OPG + AI‐PD. Using the reference clinical decision, agreement rates for clinical decisions obtained under the two information conditions (OPG‐only and OPG + AI‐PD) were calculated and compared, and the risk of overtreatment was also assessed.

**Results:**

Compared with OPG‐only, the OPG + AI‐PD condition showed higher agreement rate with the reference clinical decision, indicating that PerioAI may provide additional information for clinical decision making. Patient‐level average agreement rates increased from 77.6% to 84.7% for prognosis (*p* < 0.05) and from 78.2% to 84.3% for treatment planning (*p* < 0.05). Tooth‐level agreement rates improved from 78.1% to 86.0% for prognosis (*p* < 0.05) and from 78.8% to 85.4% for treatment planning (*p* < 0.05). The addition of AI‐PD was associated with a 42.3% reduction in overtreatment risk (Steps 1–2 vs. Step 3) and a 98.5% reduction in the risk of tooth extraction (Step 3 vs. extraction).

**Conclusions:**

When combined with radiographic information, PerioAI shows potential to provide incremental information for clinical decision making. Future research should integrate additional periodontal parameters and validate the approach in larger and more diverse populations.

## 1 Introduction

Periodontal probing and charting remain the gold standard in periodontal diagnosis (Herrera et al. 2025; Stødle et al. 2025). However, it is time consuming and highly operator‐dependent, as it is strongly linked to the clinician's training and experience (Stødle et al. 2025). Its diagnostic accuracy and reproducibility are influenced by probe angulation and insertion force (Watts 1989). Controlled‐pressure probes and electronic pressure‐sensitive systems were proposed, but their clinical advantages are limited (Mayfield et al. 1996; Renatus et al. 2016). While dentists claim to perform accurate periodontal diagnoses 95% of the time, periodontal charting is rarely performed (Ghiabi and Weerasinghe 2011; Linden et al. 1999) and is documented in the clinical record for only a minority of patients (McFall Jr et al. 1988).

Artificial intelligence (AI) is transforming medicine, especially in image analysis, diagnostic support and decision making. By utilizing deep learning and big data, AI systems improve diagnostic accuracy, consistency and efficiency compared to conventional interpretation, offering greater objectivity and scalability (Curioni‐Fontecedro 2017; Gulshan et al. 2016; Hamet and Tremblay 2017). Recent research has confirmed AI's strong performance for skin lesions, breast cancer, pulmonary nodules and diabetic retinopathy (Burlina et al. 2017; Esteva et al. 2017; Kermany et al. 2018; Poplin et al. 2018; Raju et al. 2017; Ting et al. 2017). In dentistry, AI‐based technologies have assessed alveolar bone loss on radiographs, making them suitable for disease detection (Chang et al. 2020; Chen et al. 2023; Kim et al. 2019; Krois et al. 2019; Lee et al. 2022; Li et al., 2025; Mei et al. 2024). Nevertheless, owing to the lack of soft‐tissue information and the inherent limitations of two‐dimensional imaging, they fall short of the clinical requirements for periodontal prognosis and treatment planning (Eickholz et al. 1998).

To address these limitations, our group recently developed an AI‐based diagnostic tool, PerioAI, that integrates intraoral scans (IOSs) and cone‐beam computed tomography (CBCT) images (Tan et al. 2025). By incorporating key modules such as image segmentation, multimodal registration and automated measurement, PerioAI can directly measure a digital linear distance at six standard sites per tooth (gingival margin‐to‐bone distance, GBD). These measurements can be further converted into AI‐derived probing depth (AI‐PD); however, the current system provides PD information only, and does not capture other key periodontal parameters.

In clinical practice, comprehensive periodontal charting is not always performed, and patient records often lack complete periodontal diagnostic information (Ghiabi and Weerasinghe 2011; Linden et al. 1999; McFall Jr et al. 1988). In such situations, clinicians might rely more heavily on radiographic information. Whether the additional availability of AI‐PD could provide clinically useful information remains to be investigated.

Therefore, this proof‐of‐principle study aimed to evaluate whether AI‐PD generated by PerioAI could assist clinicians in periodontal clinical decision making, including prognosis and treatment planning, when radiographic imaging represents the primary source of information.

## 2 Materials and Methods

### 2.1 Study Design, Patients and Data Collection

The study was approved by the Ethics Committee of Shanghai Ninth People's Hospital (SH9H‐2021‐T408‐3) and conducted in accordance with the Declaration of Helsinki and the WHO guidance on the ethics and governance of artificial intelligence in health (Malpani et al. 2024). It used data from subjects who underwent comprehensive periodontal examinations at Shanghai Ninth People's Hospital in the context of a registered (NCT05513599) diagnostic trial (Li, Kung, et al. 2025). The present study, therefore, used an available dataset from this ongoing diagnostic trial as a proof‐of‐principle sample.

The dataset included digital orthopantomograms (OPGs [Planmeca ProMax 2D, Planmeca Oy, Helsinki, Finland] with a resolution of 200 dpi and standard acquisition settings of 66–70 kV, 10 mA, 16 s exposure), CBCT images (NewTom VGi evo [QR s.r.l., Verona, Italy], voxel size 0.125 mm, field of view 8 × 8 cm, scanning protocol: 110 kV, 3.6 mA, 18 s exposure), intraoral scans (TRIOS 3 [3Shape, Copenhagen, Denmark], with a resolution of 20–50 μm, software version 1.7.4.5) and complete periodontal charting performed by a single trained and calibrated examiner (κ value within 1 mm of > 0.85, throughout the study) (Li, Kung, et al. 2025).

Adults with complete periodontal clinical and radiographic records and at least five natural teeth in both jaws were included if the imaging quality was sufficient for analysis (CBCT scans without severe metal artefacts, IOS data with adequate scanning range and acceptable noise interfering with the detection of gingival and crown margins and diagnostic OPGs).

To limit severe imbalance across stages of periodontitis within this proof‐of‐principle dataset, a stratified sampling approach was used to obtain a more balanced representation of disease severity in the analytical sample (Diaz‐Quijano 2018).

### 2.2 GBD Acquisition and Data Processing

PerioAI is an artificial intelligence system designed to automatically measure GBD from IOS and CBCT scans. The process of obtaining GBD using PerioAI has been previously described in detail (Tan et al. 2025). The resulting output included structured numerical GBD values and 3D visualisations (in Polygon File Format [.ply]), which could be integrated and displayed with IOS and CBCT in MeshLab (Version 2023.12). These served as the input for the validation and conversion analyses.

### 2.3 Interpretability Assessment

To assess interpretability, GBD, CBCT and IOS data were loaded into MeshLab (Version 2023.12). After automatic registration, six sites per tooth were reviewed manually to verify whether the GBD values followed clinical probing logic. Representative cases are shown in Figure S1. GBD accuracy was judged by one examiner (H.L.) based on four validated criteria (Listgarten 1980): (i) pointing to the region with the most severe bone loss; (ii) trajectory following the long axis of the tooth; (iii) close adherence to the tooth surface (no penetration); (iv) starting at the gingival margin and ending at the alveolar crest.

### 2.4 GBD to AI‐Derived Probing Depth Conversion and Periodontal Chart Construction

The difference between GBD and clinical probing depth (PD) typically ranges from 0 to 1.5 mm, as GBD measures the linear distance from the gingival margin to the alveolar bone crest while clinical probing stops within the connective tissue attachment (Schmidt et al. 2013). Consequently, to obtain AI‐derived probing depth (AI‐PD), two empirical formulas were investigated:

- Formula 1: AI‐PD = GBD − 1;
- Formula 2: AI‐PD = GBD − 0.5.

The obtained AI‐PD values were rounded to the nearest millimetre.

Only samples with a GBD–clinical PD difference of 0–2 mm were considered acceptable for exploratory evaluation and included in the analysis of conversion performance. This threshold accounts for the expected physiological discrepancy and avoids attributing PerioAI prediction errors to the formulas. The GBD values were then batch‐converted into AI‐PD and incorporated into a standardised periodontal chart format.

### 2.5 Clinical Decision‐Making Process (Case Prognosis and Treatment Plan)

Case prognosis evaluation and treatment planning were based on predefined and validated criteria (Orishko et al. 2024). Each tooth was assigned a score (Prognosis: Score 0, Hopeless; Score 1, Questionable; Score 2: Secure) and a treatment plan (Step 1: Supragingival instrumentation; Step 2: Subgingival instrumentation; Step 3: Re‐instrumentation/surgery or tooth extraction) (Sanz et al. 2020). All cases underwent three separate assessments under different information conditions, with a 2‐week interval between assessments to minimise potential recall bias. Clinical decisions were evaluated under three information conditions: OPG + original periodontal chart condition (used to establish the reference clinical decision for subsequent comparisons); OPG‐only condition; and OPG + AI‐PD condition. The time required to complete each assessment was recorded (in seconds). All assessments were performed by a single certified periodontist (A.R.). Intra‐examiner reliability was evaluated, with methodological details provided in Supporting Information.

### 2.6 Data Analysis

Bland–Altman analysis was used as a descriptive approach to illustrate the agreement between GBD generated by the PerioAI system and clinical PD, as well as between AI‐PD and clinical PD. For formulas converting GBD into PD, the performance was evaluated using the coefficient of determination (*R* ^2^), absolute accuracy (proportion of sites with exact agreement between AI‐PD and clinical PD) and relative accuracy (proportion of sites with a difference within ±1 mm). For clinical decision making (prognosis and treatment plan), the assessment based on the original periodontal chart + OPG was used to establish the reference for subsequent comparisons. Using the reference clinical decision as the comparator, agreement rates were calculated for clinical decisions under the OPG‐only condition and OPG + AI‐PD conditions. Agreement rates were evaluated both at the patient level (defined as the proportion of teeth with consistent decisions relative to the total number of teeth per patient) and at the tooth level (defined as the proportion of teeth with consistent decisions relative to the total number of teeth within each information condition). Detailed procedures are provided in Supporting Information. Relative risk (RR) and the corresponding 95% confidence interval (CI) were calculated to compare the likelihood of overestimating treatment needs between the OPG + AI‐PD and OPG‐only conditions. Finally, the time required (in seconds) for assessing each case was recorded. Differences between conditions were evaluated using independent two‐sample *t*‐tests and one‐way analysis of variance (ANOVA). All analyses were conducted in Python (v3.10) using scipy.stats, statsmodels, sklearn.metrics and matplotlib. Significance was set at *p* < 0.05.

## 3 Results

### 3.1 Patient and Tooth Characteristics

The study included 53 participants (28 males, *p* = 0.418; mean age: 41.43 ± 11.34 years, range: 20–68 years). Participant, tooth/site distribution and clinical characteristics are summarised in Table 1.

**TABLE 1:** Demographic characteristics, periodontal status and clinical parameters of study participants (*n* = 53).

	Total	G + Stage I	Stage II	Stage III	Stage IV	
Demographics						
Male	28	4	7	12	5	
Female	25	7	8	6	4	
Total	53	11	15	18	9	
Age, years						
18–29	7	4	2	0	1	
30–59	41	7	12	17	5	
≥ 60	5	0	1	1	3	
Smoking status						
Never	42	11	11	13	7	
Former	4	0	2	2	0	
Current	7	0	2	3	2	
Number of teeth						
Number of single‐rooted teeth	964	217	278	330	139	
Number of multiple‐rooted teeth	334	73	97	115	49	
Number of total teeth	1298	290	375	445	188	
Number of sites						
PD ≤ 3 mm	5310	1563	1498	1782	467	
PD = 4–5 mm	1744	165	545	717	317	
PD ≥ 6 mm	734	12	207	171	344	
Clinical parameters						
PD (mean ± SD)	3.46 ± 1.53	2.62 ± 0.77	3.28 ± 1.19	3.37 ± 1.22	4.62 ± 2.22	
CAL (mean ± SD)	2.83 ± 2.52	0.45 ± 0.65	1.41 ± 1.48	3.15 ± 1.89	5.70 ± 2.69	
Furcation involved teeth (Classes II and III) per patient (mean ± SD)	1.74 ± 2.12	ND	ND	2.61 ± 1.88	3.56 ± 1.88	
Mobile teeth (grade II & III) per patient (mean ± SD)	1.38 ± 3.76	ND	ND	0.50 ± 0.86	6.33 ± 7.33	
BOP percentage per patient (mean ± SD)	66.46% ± 17.69%	52.26% ± 23.11%	66.45% ± 16.35%	70.64% ± 11.87%	76.57% ± 15.32%	

Abbreviations: BOP, bleeding on probing percentage; CAL, clinical attachment loss; ND, not detected; PD, probing depth.

### 3.2 Evaluation of GBD Interpretability

At the patient level, 33.9% of individuals showed errors, with a significant increase in prevalence as periodontal status worsened (G + Stage I: 9.1% [95% CI: 1.6%–37.7%]; Stage IV: 66.7% [95% CI: 35.4%–87.9%]) (*p* < 0.05). At the tooth level, 55 out of 1298 teeth (4.2% [95% CI: 3.3%–5.5%]) exhibited errors, with multi‐rooted teeth displaying a significantly higher error rate (6.9%, 23/334 [95% CI: 4.6%–10.1%] vs. 3.3%, 32/964 [95% CI: 2.4%–4.6%], *p* < 0.001). Similar trends were observed regarding probing depths (shallow vs. deep pockets) and locations (inter‐dental vs. buco‐lingual sites) (*p* < 0.001). Figure S1 depicts a representative error case, while Table S1 summarises the distribution of errors across different groups. Further details are provided in Supporting Information.

### 3.3 Agreement Between GBD and Clinical PD, Conversion Formula From GBD to AI‐PD and Agreement Between AI‐PD and Clinical PD

**FIGURE 1:**
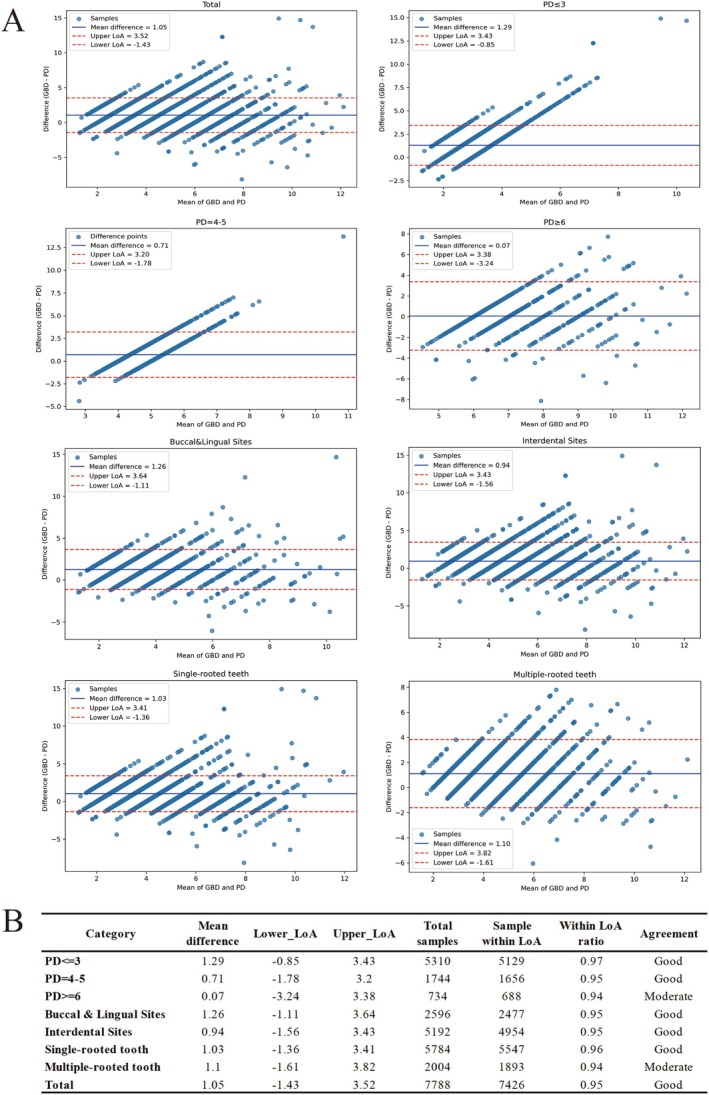
Agreement between gingiva‐to‐bone distance (GBD) and clinical probing depth (PD) across subgroups. (A) Bland–Altman plots showing the agreement between GBD and PD across eight clinical subgroups: Sites with PD ≤ 3 mm, PD = 4–5 mm and PD ≥ 6 mm; buccal and lingual sites; interdental sites; single‐rooted tooth; multiple‐rooted tooth; and overall. (B) Agreement was classified as ‘Good’ when ≥ 95% of samples are within limits of agreement (LoA) and as ‘Moderate’ otherwise.

A moderate‐to‐good agreement between GBD and PD measurements was observed across eight periodontal assessment categories. Figure 1A shows systematic trends in which differences between methods vary with measurement size, indicated by non‐zero mean differences (ranging from 0.07 to 1.29) and varying limits of agreement (LoAs). Most categories exhibited proportional bias, with differences increasing as average values grew, especially in total, buccal and lingual sites and multirooted teeth plots. Figure 1B quantifies this agreement, showing that 94%–97% of measurements fell within the LoA across all categories, with ratios consistently between 0.94 and 0.97. Although mean differences were relatively small (0.07–1.29), the wide LoA range (around 5 mm) suggests clinically significant variability between the two methods, particularly for deep pockets.

**FIGURE 2:**
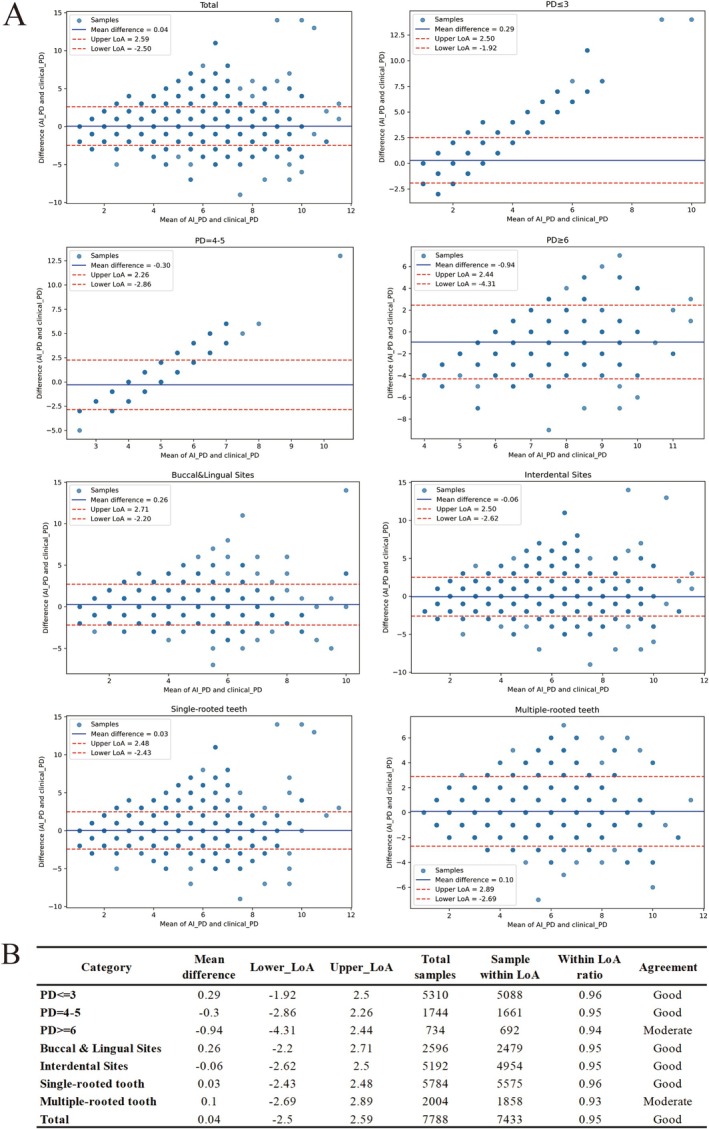
Agreement between AI‐PD and clinical PD across subgroups (based on all sites, including those with incorrect GBD predictions). (A) Bland–Altman plots showing the agreement between AI‐PD and clinical PD across eight clinical subgroups: Sites with PD ≤ 3 mm, PD = 4–5 mm and PD ≥ 6 mm; buccal and lingual sites; interdental sites; single‐rooted tooth; multiple‐rooted tooth; and overall. (B) Summary statistics for each subgroup, including mean difference, 95% limits of agreement (LoA) and proportion of samples within LoA. Agreement was classified as ‘Good’ when ≥ 95% of samples fell within LoA and as ‘Moderate’ otherwise.

Between the two tested conversion formulas, GBD‐0.5 demonstrated superior performance, with higher absolute (43%–52%) and relative accuracy (89%–96%) than GBD‐1 (34%–51% and 100%, respectively), particularly in deep pockets. Both formulas demonstrated moderate to strong predictive power, with *R* ^2^ values of 0.70 for GBD‐1 and 0.57 for GBD‐0.5, indicating that GBD‐1 explains more variance, despite lower absolute accuracy metrics. Details of the conversion formulas and of the Bland–Altman analysis are provided in Table 2 and Figure 2.

**TABLE 2:** Performance of two formulas for converting GBD to PD across different clinical subgroups (based on sites with accurate GBD predictions).

	Absolute accuracy		Relative accuracy		*R* ^2^	
	AI‐PD = GBD – 1	AI‐PD = GBD – 0.5	AI‐PD = GBD – 1	AI‐PD = GBD – 0.5	AI‐PD = GBD – 1	AI‐PD = GBD – 0.5
All Samples	47%	43%	100%	91%	0.70	0.57
Probing depth ≤ 3	51%	40%	100%	89%		
Probing depth = 4–5	38%	52%	100%	95%		
Probing depth ≥ 6	34%	48%	100%	96%		

*Note*: AI‐PD refers to PD values calculated from GBD (gingival margin‐to‐bone distance). Performance metrics include the following: Absolute accuracy: proportion of sites where the AI‐PD exactly matches the clinical PD; Relative accuracy: proportion of sites where the difference between AI‐PD and clinical PD is within ±1 mm; Coefficient of determination (*R* ^2^).

The overall agreement was rated as ‘Good’ for most categories and ‘Moderate’ for PD ≥ 6 and multiple‐rooted teeth. In addition, 85.1% of sites (6631/7788) showed an absolute difference between AI‐PD and clinical PD within ±1 mm.

### 3.4 Case Assessment (Tooth Prognosis and Treatment Planning)

The intra‐examiner reliability exceeded 95% for both tooth prognosis and treatment planning across all evaluation pathways. Specifically, intra‐examiner agreement was as follows: OPG + original periodontal chart: prognosis: 1.000, treatment plan: 0.995; OPG + AI‐PD: prognosis: 0.981, treatment plan: 0.992; OPG‐only: prognosis: 0.966, treatment plan: 0.987.

Results for prognosis and treatment plan assessment at the patient level are shown in Table 3 (additional clarifications are given in Supporting Information). It shows the agreement rate between the reference clinical decision (based on the original periodontal chart + OPG) and the clinical decisions made under the OPG‐only condition and OPG + AI‐PD conditions. Agreement rates with the reference clinical decision were higher under the OPG + AI‐PD condition than under the OPG‐only condition. Table 4 presents the corresponding tooth‐level analyses. Under the OPG + AI‐PD condition, significantly higher overall agreement rates were observed at the tooth level compared with the OPG‐only condition (86.0% vs. 78.1%, *p* < 0.05). An improvement was also observed for teeth with shallow pockets (PD ≤ 3), where agreement increased from 92.8% to 96.8% (*p* < 0.05). Higher agreement rates were observed for single‐rooted teeth than for multiple‐rooted teeth across probing depth categories.

**TABLE 3:** Patient‐level agreement rate of prognosis and treatment planning with the reference clinical decision under the OPG‐only and OPG + AI‐PD conditions.

(A) Prognosis at patient level							
Group	Total cases	≥ 80% (no./%)			Mean agreement rate		
		OPG‐only	OPG + AI‐PD	*p*	OPG‐only	OPG + AI‐PD	*p*
G + Stage I	11	11 (100%)	10 (90.9%)	1	97.30%	95.60%	0.176
Stage II	15	7 (46.7%)	12 (80.0%)	0.128	75.90%	85.60%	0.1
Stage III	18	5 (27.8%)	10 (55.6%)	0.176	72.90%	80.80%	0.059
Stage IV	9	4 (44.4%)	5 (55.6%)	1	65.50%	77.70%	0.203
Overall	53	27 (50.9%)	37 (69.8%)	0.073	77.60%	84.70%	< 0.05

(B) Treatment plan at patient level							
Group	Total cases	≥ 80% (no./%)			Mean agreement rate		
		OPG‐only	OPG + AI‐PD	p	OPG‐only	OPG + AI‐PD	p
G + Stage I	11	11 (100%)	10 (90.9%)	1	97.70%	95.60%	0.08
Stage II	15	8 (53.3%)	12 (80.0%)	0.245	78.50%	85.10%	0.182
Stage III	18	6 (33.3%)	11 (61.1%)	0.181	71.90%	80.50%	< 0.05
Stage IV	9	5 (55.6%)	4 (44.4%)	1	66.60%	75.30%	0.332
Overall	53	30 (56.6%)	37 (69.8%)	0.227	78.20%	84.30%	< 0.05

*Note*: (A) Prognosis: Agreement rates for prognostic classification across subgroups. Score 0 (Hopeless), Score 1 (Questionable), Score 2 (Secure). (B) Treatment plan: Agreement rate in treatment plan across patient subgroups. Step 1–2 (supragingival and subgingival instrumentation), Step 3 (re‐instrumentation/surgery) and tooth extraction. See Supporting Information for detailed explanation of the calculations and testing.

**TABLE 4:** Tooth‐level agreement rate of prognosis and treatment planning with the reference clinical decision under the OPG‐only and OPG + AI‐PD conditions.

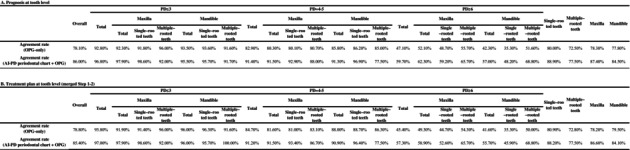

*Note*: Agreement rates were calculated at the tooth level using the OPG + original periodontal chart as the reference standard and are reported for the two information conditions (OPG‐only and OPG + AI‐PD) across subgroups. (A) Prognosis: Agreement rates for prognostic classification across subgroups. Score 0 (Hopeless), Score 1 (Questionable), Score 2 (Secure). (B) Treatment plan: Agreement rates in treatment plan across patient subgroups. Step 1–2 (supragingival and subgingival instrumentation), Step 3 (re‐instrumentation/surgery) and tooth extraction.

Tooth‐level treatment planning showed similar trends, with overall agreement rates increasing from 78.8% under the OPG‐only condition to 85.4% under the OPG + AI‐PD condition (*p* < 0.05). Improvements were observed in teeth with deep pockets (PD ≥ 6), where agreement increased from 45.4% to 57.3% (*p* < 0.05), including mandibular teeth (50.0%–68.8% for multiple‐rooted teeth, *p* < 0.05). Shallow pockets (PD ≤ 3) showed an increase from 93.8% to 97.0% (*p* = 0.0161), and moderate pockets (PD = 4–5) increased from 84.7% to 91.2% (*p* < 0.05) (Table 4). Given the small subgroup sample size, these subgroup findings should be interpreted cautiously and considered hypothesis‐generating only.

**FIGURE 3:**
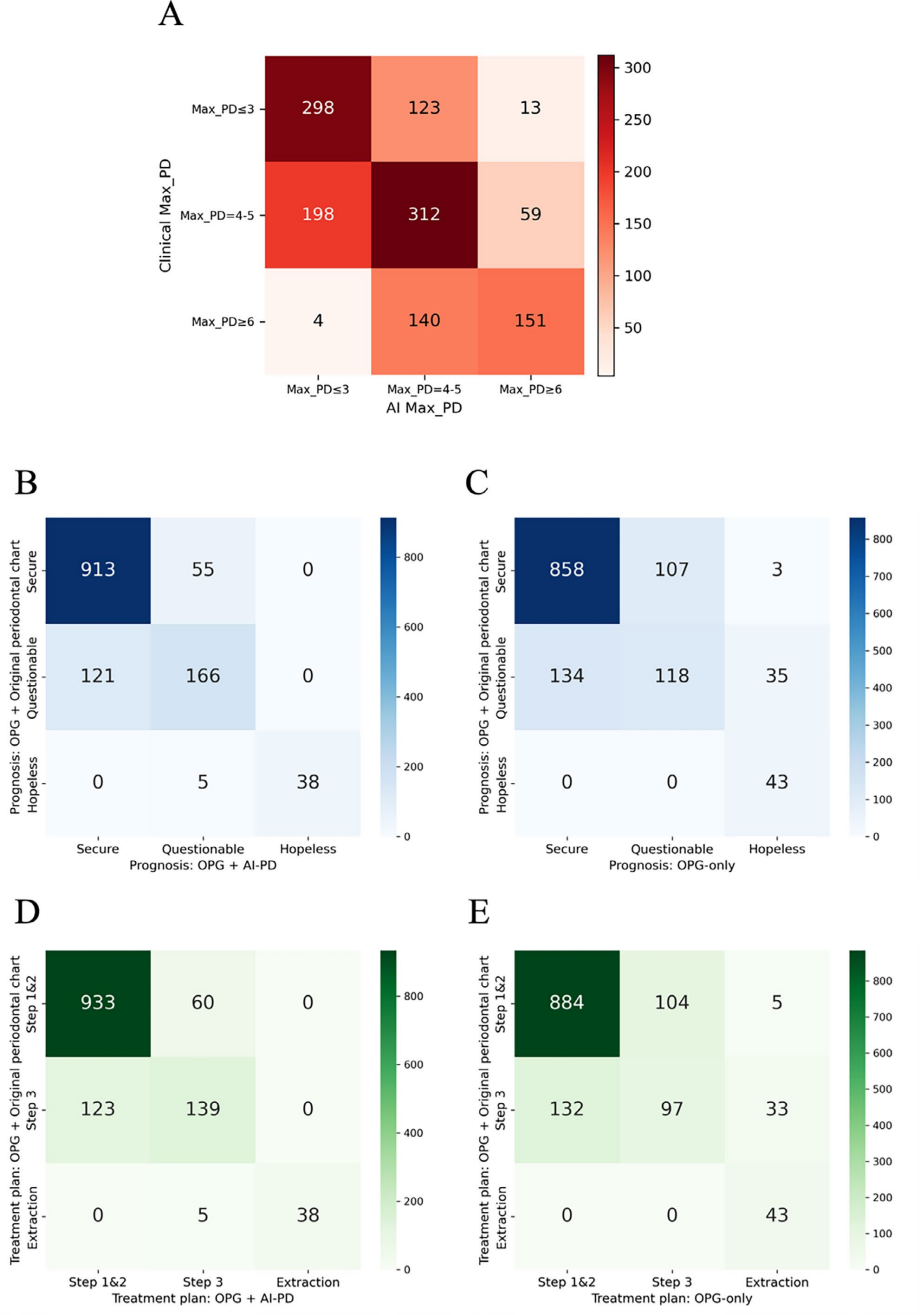
Tooth‐level confusion matrices comparing clinical charting and AI‐derived probing depths, prognosis and treatment planning. (A) Confusion matrix comparing AI‐generated maximal PD for the individual tooth (Max_PD vs. clinical Max_PD at the tooth level). (B) Confusion matrix for prognosis comparing the reference clinical decision (OPG + original periodontal chart) with the OPG + AI‐PD condition. (C) Confusion matrix for prognosis comparing the reference clinical decision (OPG + original periodontal chart) with the OPG‐only condition. (D) Confusion matrix for treatment planning comparing the reference clinical decision (OPG + original periodontal chart) with the OPG + AI‐PD condition. (E) Confusion matrix for treatment planning comparing the reference clinical decision (OPG + original periodontal chart) with the OPG‐only condition. Clinical Max‐PD refers to the maximum probing depth of each tooth recorded in the original periodontal chart, while AI Max‐PD refers to that generated in the AI‐PD periodontal chart. Treatment plan: Step 1–2 (supragingival + subgingival instrumentation), Step 3 (re‐instrumentation/surgery), extraction. PD thresholds for decision making are according to Sanz et al. (2020). The reference standard dataset included bleeding on probing, attachment levels, furcation involvement and mobility, which were not available in the AI‐PD dataset.

The relative risk (RR) of overestimating Step 1 + 2 as 3 was 0.577 (95% CI: 0.42–0.78) when using OPG + AI‐PD condition compared to the OPG‐only condition, while it was 0.015 (95% CI: 0.001–0.25) for overestimating the need for Step 3 as tooth extraction when AI‐PD periodontal chart + OPG was used. Figure 3 shows the confusion matrices between the highest AI‐PD (AI Max_PD) and its clinical equivalent (clinical Max_PD) at the tooth level, along with the corresponding clinical decision outcomes. Regarding the time needed for prognosis and treatment planning, relying solely on OPG was faster than AI‐PD coupled with OPG and complete periodontal charting with OPG. Additionally, more advanced cases took more time (Table S2). Figure 3 shows the confusion matrices.

## 4 Discussion

This proof‐of‐principle study was designed to assess the potential of PerioAI, an AI‐based system that combines IOSs and CBCT to automatically measure the gingival margin‐to‐bone distance (GBD), which can subsequently be converted into AI‐derived probing depth (AI‐PD). Specifically, the present study explored the potential role of AI‐PD in periodontal clinical practice. The results indicate that although PerioAI currently provides only a single parameter (AI‐PD), which remains imperfect, adding AI‐PD information improved agreement between clinical decisions (prognosis and treatment planning) and the reference clinical decision compared with the OPG‐only condition. In addition, incorporating AI‐PD information was associated with a lower likelihood of overtreatment and unnecessary tooth extraction suggestions. These findings suggest that, once further developed and validated, AI‐based methods may provide additional information in situations where comprehensive periodontal diagnostic information is not consistently documented in routine clinical records (Ghiabi and Weerasinghe 2011; Linden et al. 1999; McFall Jr et al. 1988).

When examining the performance of the AI system in more detail, only 3.1% of sites (239/7788) showed obvious errors in GBD measurement, meaning that the automatically generated measurement path or landmarks were judged to be clearly inconsistent with the expected anatomical measurement trajectory, for example, when the trajectory did not follow the tooth axis, or the endpoints did not correspond to the gingival margin and alveolar crest. These findings suggest that the GBD measurements generated by PerioAI generally comply with predefined anatomical criteria and therefore demonstrate a certain level of interpretability. However, the current version of the model still requires further refinement, particularly in cases with more severe periodontal lesions. In such situations, deeper pockets are frequently associated with intrabony and inter‐radicular defects, and these complex anatomical conditions may complicate AI systems' identification of alveolar bone morphology, resulting in deviations in the position or orientation of the GBD measurement trajectory (Tan et al. 2025).

Similarly, Bland–Altman analyses (clinical PD vs. GBD and clinical PD vs. AI‐PD) showed the same trend, with LoA widening as the probing depth increased. This observation is consistent with the earlier finding that measurement errors occurred more frequently in sites with more severe periodontal lesions. LoA extending to several millimetres indicates substantial deviation and represents an important limitation of the current method. Therefore, the agreement observed here remains insufficient to support replacing conventional periodontal probing, particularly in deeper sites and in the presence of complex anatomical defects.

Clinically, it is vital to assess whether this uncertainty might impact decisions. In periodontal practice, PD is mainly used to categorise periodontal pockets into depth ranges (1–3 mm: shallow pockets; 4–5 mm: moderate pockets; ≥ 6 mm: deep pockets) to aid in disease staging and treatment planning. Consequently, confusion matrices comparing AI‐PD with clinical PD were constructed across these depth categories.

Misclassification of shallow or moderate pockets as deep was relatively uncommon, accounting for 3% (13/434) and 10% (59/569), respectively. Although this could lead to more aggressive treatment recommendations, it did not result in erroneous extraction decisions when clinical decisions were made under the OPG + AI‐PD condition. In contrast, cases of inappropriate extraction recommendations were observed under the OPG‐only condition. These findings suggest that even when AI‐PD contains measurement errors, it is unlikely to independently lead to erroneous extraction decisions. The extent of bone loss visible on radiographs provides an important reference for interpreting PD estimates and may help clinicians to identify implausibly deep measurements. Overall, the information provided by OPG and AI‐PD appears complementary, and their combined use may reduce the likelihood of erroneous extraction decisions. It should also be noted that when AI‐PD underestimates periodontal pocket depth, there is a potential risk of undertreatment, particularly in cases where sites that would normally require Step 2 therapy might instead receive only Step 1 treatment. In addition, clinical decisions under the OPG + AI‐PD condition showed higher agreement with the reference assessment than those under the OPG‐only condition, increasing from 78% to 86% for tooth prognosis and from 79% to 85% for treatment planning.

This study has several important limitations. First, these findings should be interpreted as exploratory observations derived from decision scenarios constructed under restricted information conditions, rather than as a complete simulation of periodontal diagnosis. Specifically, the current version of PerioAI provides only a single parameter, namely AI‐derived probing depth (AI‐PD), and cannot capture several key clinical variables required for comprehensive periodontal diagnosis, including bleeding on probing (BOP), clinical attachment level, gingival recession, furcation involvement, tooth mobility, suppuration, plaque and gingival phenotype. Because periodontal clinical decision making typically requires integrating multiple clinical parameters, the absence of these variables constitutes a fundamental limitation.

Second, the sample size was relatively small. After stratification by disease severity, subgroup sizes became even smaller, which may reduce statistical power and lead to unstable performance estimates. Therefore, the subgroup analyses presented here should be interpreted cautiously as exploratory and hypothesis‐generating observations limited to this study population rather than findings that can be generalised to broader clinical settings.

Third, this was a cross‐sectional, single‐centre study, which limits the ability to evaluate PerioAI's performance in detecting disease progression or monitoring treatment response. In addition, the clinical case assessments were performed by a single periodontist, which may also influence the external validity of the findings.

Finally, the measurement accuracy of AI‐PD still requires further improvement. Future development of PerioAI should aim to enhance measurement precision, reduce estimation errors and integrate additional clinically relevant diagnostic variables in order to better approximate a comprehensive periodontal assessment. Ideally, such systems should be validated in larger, multi‐centre cohorts including patients across a wider spectrum of periodontal disease. Moreover, the current framework relies on CBCT imaging, and future research should explore lower radiation or radiation‐free imaging approaches to improve clinical feasibility. The economic implications of using three‐dimensional imaging in periodontal assessment should also be systematically evaluated through cost‐effectiveness analyses.

Conceptually, this study provides a framework for the future development and evaluation of periodontal diagnostic technologies. On one hand, it is important to pursue high analytical precision and further optimise models to minimise measurement errors and improve data quality. On the other hand, the clinical context and the real‐world consequences of misclassification should also be carefully considered when evaluating new diagnostic approaches. In periodontal practice, clinical decision making typically relies on multiple sources of information, and replacing established reference standards, such as periodontal probing, is a substantial challenge. Nevertheless, the findings of this proof‐of‐principle study suggest that incorporating AI‐PD alongside radiographic information may provide incremental information compared with relying solely on radiographic imaging.

## 5 Conclusion

When combined with radiographic information, PerioAI‐derived probing depth may provide incremental information to support periodontal clinical decision making and has shown improved agreement with reference assessments compared with radiographic information alone. As an early proof‐of‐principle study, these findings should be interpreted as exploratory and hypothesis‐generating. Future research should integrate additional periodontal parameters and validate the approach in larger and more diverse populations.

Supporting Information includes detailed methodological procedures, clarification of agreement rate calculations and additional analyses on GBD measurement errors and evaluation time. These materials provide further transparency and support for the main findings and are available online.

## Author Contributions

Hairui Li and Yuan Li contributed to study design, data collection, analysis and interpretation and manuscript preparation. Minhui Tan and Zhiming Cui contributed to data analysis and interpretation. D.S. contributed to study design and data analysis. Andrea Roccuzzo contributed to data collection, analysis and interpretation, and led the manuscript preparation. Maurizio S. Tonetti devised this study and contributed to protocol development, data interpretation and manuscript preparation. All authors gave their final approval and agreed to be accountable for all aspects of the work.

## Funding

This research was supported by the National Natural Science Foundation of China (grant numbers 82301157, 6230012077, U23A20295, 62131015 and 62476033) and the Cross‐Disciplinary Research Fund of Shanghai Ninth People's Hospital, Shanghai Jiao Tong University School of Medicine (JYJC202135).

## Conflicts of Interest

Maurizio S. Tonetti has received grant support and/or personal fees from Geistlich Pharma AG (Switzerland), Straumann AG (Switzerland) and Nobel Biocare SA (Sweden), which are unrelated to the present work. Yuan Li, Minhui Tan, Zhiming Cui, Dinggang Shen and Maurizio S. Tonetti have submitted a patent application on Perio‐AI. The other authors declare no conflicts of interest.

## Supporting information


**Data S1:** Supporting information.
**Figure S1:** Example of a GBD measurement error observed during interpretability review: The GBD vector (shown as two green lines) incorrectly originates from the edge of the missing part caused by crown segmentation from the IOS (yellow dashed line) rather than from the gingival margin (red dashed line).
**Table S1:** Distribution of GBD positional and angular errors across patients, teeth and sites.
**Table S2:** Comparison of evaluation time based on three methods.

## Data Availability

The data that support the findings of this study are available on request from the corresponding author. The data are not publicly available due to privacy or ethical restrictions.
