# Supplementary material for: Exploring the Potential of the PerioAI System to Support Periodontal Clinical Decision Making: A Proof‐of‐Principle Study
Source: J Clin Periodontol. 2026 May 3;53(7):1056–67. doi: 10.1111/jcpe.70138 (PMC13263713; doi:10.1111/jcpe.70138)
Supplement: Supplementary file 1 — Data S1: Supporting information. Figure S1: Example of a GBD measurement error observed during interpretability review: The GBD vector (shown as two green lines) incorrectly originates from the edge of the missing part caused by crown segmentation from the IOS (yellow dashed line) rather than from the gingival margin (red dashed line). Table S1: Distribution of GBD positional and angular errors across patients, teeth and sites. Table S2: Comparison of evaluation time based on three methods. [file JCPE-53-1056-s001.docx]

**Supplementary Materials to:**

**Exploring the potential of the PerioAI system to support periodontal clinical decision-making:**

**a proof-of-principle study**

Hairui Li^1,2^*, Yuan Li^1,2^*, Minhui Tan^3^, Zhiming Cui^3^, Dinggang Shen^3,4,5^,

Andrea Roccuzzo^1,2,6^**, Maurizio S. Tonetti^1,2,7^**

^1^Shanghai Perio-Implant Innovation Center, Institute of Integrated Oral, Craniofacial and Sensory Research, Shanghai Ninth People’s Hospital, Shanghai Jiao Tong University School of Medicine, Shanghai, China

^2^College of Stomatology, Shanghai Jiao Tong University, National Center of Stomatology, National Clinical Research Center for Oral Diseases, Shanghai Key Laboratory of Stomatology, Shanghai, China

^3^School of Biomedical Engineering & State Key Laboratory of Advanced Medical Materials and Devices, ShanghaiTech University, Shanghai, China

^4^Shanghai United Imaging Intelligence Co., Ltd., Shanghai, China

^5^Shanghai Clinical Research and Trial Center, Shanghai, China

^6^Department of Periodontology, School of Dental Medicine, University of Bern, Bern, Switzerland

^7^European Research Group on Periodontology, Genova, Italy

^*^Hairui Li and Yuan Li share first author position

^**^Andrea Roccuzzo and Maurizio Tonetti share senior author position

**Correspondence**:

Maurizio S. Tonetti, Perio-Implant Innovation Center, 4F Building 1, 115 Jinzun Road, Pudong Research Campus, Shanghai Jiao Tong University School of Medicine, 200115, Shanghai, China.

email: maurizio.tonetti@ergoperio.eu

**Appendix**

**Supplementary Methods:**

**Detailed procedure for the assessment of intra-examiner reliability**

To assess intra-examiner reliability, after completion of the main analysis, a stratified random sample was drawn from the 53 patients according to disease stage (Stages I–IV), with three patients selected from each stage (total n = 12) to ensure representation across the spectrum of disease severity in the repeat assessment. The same examiner then reclassified all teeth of the selected patients under blinded conditions and in random order using the same three interpretation conditions. A minimum two-week interval was applied between repeated assessments to minimize potential recall bias.

**Clarifications of the calculations in Table 3 and 4 of the main text.**

**Patient-level agreement rate**

Using prognosis assessment as an example, consider a patient with 28 teeth. Prognostic judgments for all 28 teeth derived from *OPG combined with conventional periodontal charting* were used as the reference standard. Subsequently, for the same patient, the clinician performed a second prognostic assessment of the same 28 teeth based on *OPG combined with the AI-PD periodontal chart*, and each tooth-level prognosis was compared with the reference standard.

For instance, if prognostic judgments for 21 out of 28 teeth were concordant with the reference standard, the agreement rate for that patient under this assessment pathway would be 21/28, i.e., 75%.

**Interpretation of the “≥80% (num/%)” metric**

This metric was used to describe the proportion of patients within a given disease stage whose agreement rate reaches or exceeds 80%. For example, if a disease stage group (e.g., Stage I) consists of three patients, and their patient-level agreement rates under a given assessment pathway are calculated individually, with two patients achieving agreement rates of 81% and 83% (both ≥80%) and the third patient having an agreement rate below 80%, then the value of “≥80% (num/%)” for that stage group would be 2 (66.7%), corresponding to 2 out of 3 patients.

It should be emphasized that this metric was intended solely to describe the proportion of patients with high agreement within the group, and it was not used as a threshold for result selection or system inclusion.

**Calculation of the mean agreement rate**

The mean agreement rate was defined as the arithmetic mean of the patient-level agreement rates across all patients within a given disease stage group, including all patients regardless of whether their agreement rate reaches the 80% threshold. For example, if a Stage I group includes three patients with agreement rates of 25%, 50%, and 75%, respectively, the mean agreement rate for this group would be (25% + 50% + 75%)/3 = 50%. Agreement rates below 80% are fully included in the calculation and are not excluded.

**Statistical testing applied in Table 3.**

Statistical comparisons in Table 3 were conducted under the following two scenarios:

1. Comparison between *OPG-only* and *OPG + AI-PD* with respect to the “≥80% (num/%)” metric, with *p* values were calculated using Fisher’s exact test.

2. Comparison between *OPG-only* and *OPG + AI-PD* with respect to the mean agreement rate, with *p* values were calculated using the Wilcoxon signed-rank test.

**Proposed “added value” metric of PerioAI.**

It should be clarified that the 80% agreement threshold was neither a criterion for system output nor a prerequisite for statistical significance. The added value of PerioAI does not lie in excluding predictions with lower agreement, but rather in the observation that, within the same patient population, the introduction of AI-PD—compared with the use of OPG alone—leads to higher patient-level mean agreement rates and an increased proportion of patients achieving high agreement (≥80%).

**Supplementary Results:**

**Figure S1.** **Example of a GBD measurement error observed during interpretability review: The GBD vector (shown as two green lines) incorrectly originates from the edge of the missing part caused by crown segmentation from the IOS (yellow dashed line), rather than from the gingival margin (red dashed line).**


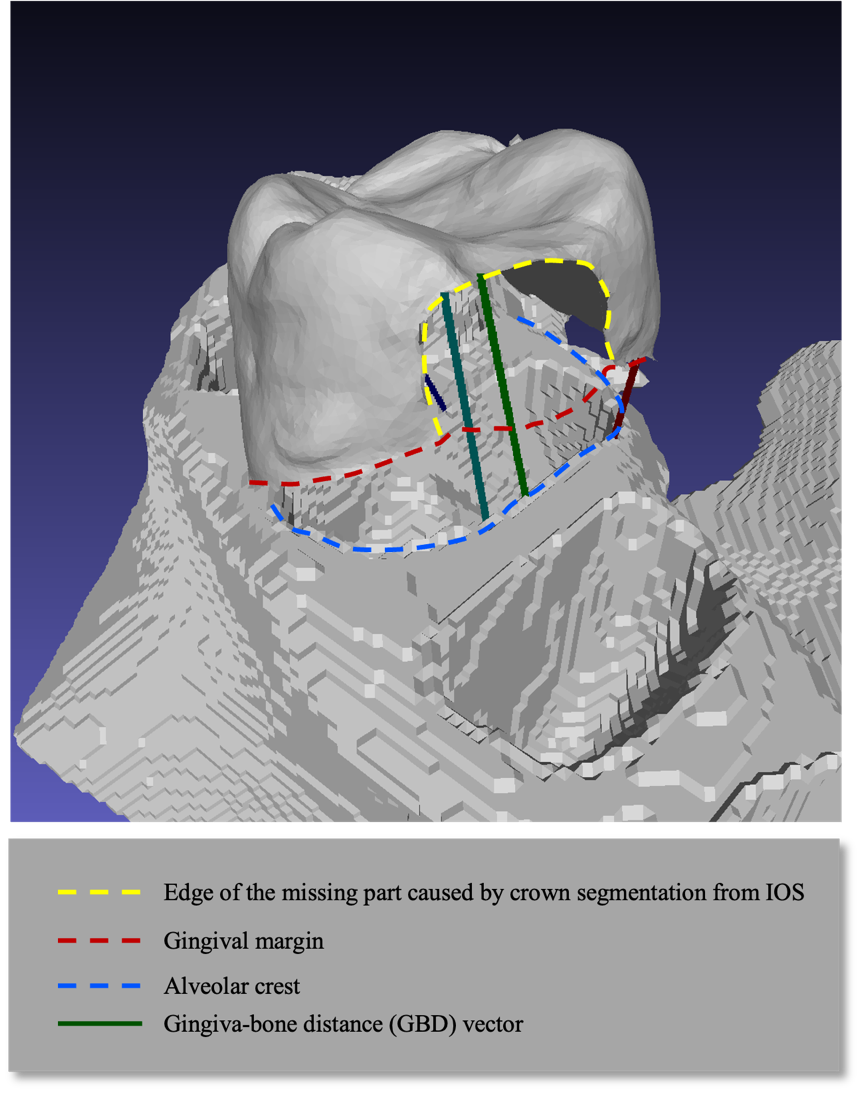


| **Table S1. Distribution of GBD positional and angular errors across patients, teeth, and sites** | | | | | | | |
| --- | --- | --- | --- | --- | --- | --- | --- |
|  |  | **Total** | **G +Stage 1** | **Stage 2** | **Stage 3** | **Stage 4** | **p-Value** |
| **Number of patients** |  | 18/53 (33.9%) | 1/11 (9.1%) | 4/15 (26.7%) | 7/18 (38.9%) | 6/9 (66.7%) | < 0.05 |
| **Number of teeth** |  |  |  |  |  |  | < 0.001 |
|  | **Single-rooted tooth** | 32/964 (3.3%) | 1/217 (0.5%) | 2/278(0.7%) | 13/330 (3.9%) | 16/139 (11.5%) |  |
|  | **Multiple-rooted tooth** | 23/334 (6.9%) | 0/73 (0%) | 4/97 (4.1%) | 10/115 (8.7%) | 9/49 (18.4%) |  |
|  | **Total** | 55/1298 (4.2%) | 1/290 (0.3%) | 6/375 (1.6%) | 23/445 (5.2%) | 25/188 (13.3%) |  |
| **Number of Sites** |  |  |  |  |  |  | < 0.001 |
|  | **Single-rooted tooth** | 145/5784 (2.5%) | 6/1302(0.5%) | 12/1668 (0.7%) | 57/1980 (2.9%) | 70/834 (8.4%) |  |
|  | **Multiple-rooted tooth** | 94/2004 (4.7%) | 0/438 (0%) | 24/582 (4.1%) | 28/690 (4.1%) | 42/294 (14.3%) |  |
|  | **Total** | 239/7788 (3.1%) | 6/1740 (0.3%) | 36/2250 (1.6%) | 85/2670 (3.2%) | 112/1128 (9.9%) |  |
|  |  |  |  |  |  |  | < 0.001 |
|  | **PD≤3** | 122/5310 (2.3%) | 6/ 1563 (0.4%) | 22/1498 (1.5%) | 58/1782 (3.3%) | 36/467 (7.7%) |  |
|  | **PD=4-5** | 44/ 1744 (2.5%) | 0/165 (0%) | 10/545 (1.8%) | 15/717 (2.1%) | 19/317 (5.9%) |  |
|  | **PD≥6** | 73/734 (9.9%) | 0/12 (1%) | 4/207 (1.9%) | 12/171 (7.0%) | 57/344 (16.6%) |  |
|  | **Total** | 239/7788 (3.1%) | 6/1740 (0.3%) | 36/2250 (1.6%) | 85/2670 (3.2%) | 112/1128 (9.9%) |  |
|  |  |  |  |  |  |  | < 0.001 |
|  | **Buccal & Lingual Sites** | 69/2596 (2.7%) | 2/580(3.4%) | 12/750 (1.6%) | 22/890 (2.5%) | 33/376 (8.8%) |  |
|  | **Interdental Sites** | 170/5192 (3.3%) | 4/1160 (0.3%) | 24/1500 (1.6%) | 63/1780 (3.5%) | 79/752 (10.5%) |  |
|  | **Total** | 239/7788 (3.1%) | 6/1740 (0.3%) | 36/2250 (1.6%) | 85/2670 (3.2%) | 112/1128 (9.9%) |  |

Categorical data are reported as counts (n) and percentages. Statistical comparisons across groups were performed using Chi-square tests. GBD = Gingiva-bone distance; G = Gingivits; PD = Probing depth

**Table S2. Comparison of evaluation time based on three methods.**

| **Group** | **Count** | **Original periodontal chart + OPG** | | **AI-PD periodontal chart + OPG** | | **OPG-only** | | **p-value**  **(Original periodontal chart + OPG**  **vs**  **AI-PD periodontal chart + OPG)** | **p-value**  **(Original periodontal chart + OPG**  **vs**  **OPG-only)** | **p-value**  **(AI-PD periodontal chart + OPG**  **vs**  **OPG-only)** |
| --- | --- | --- | --- | --- | --- | --- | --- | --- | --- | --- |
|  |  | **Mean±SD（seconds）** | **95% CI** | **Mean±SD（seconds）** | **95% CI** | **Mean±SD（seconds）** | **95% CI** |  |  |  |
| P1+G | 4 | 191.5±37.62 | (131.64, 251.36) | 152.25±31.55 | (102.04, 202.46) | 70.25±29.16 | (23.85, 116.65) | 0.161 | <0.05 | <0.05 |
| P2 | 15 | 225.2±71.13 | (185.81, 264.59) | 177.47±53.26 | (147.97, 206.96) | 113.6±57.13 | (81.96, 145.24) | <0.05 | <0.05 | <0.05 |
| P3 | 18 | 303.61±100.05 | (253.86, 353.36) | 229.22±77.15 | (190.86, 267.59) | 120±42.48 | (98.87, 141.13) | <0.05 | <0.05 | <0.05 |
| P4 | 9 | 260.67±85.68 | (194.81, 326.52) | 281.67±105.07 | (200.9, 362.43) | 131.67±36.51 | (103.6, 159.73) | 0.648 | <0.05 | <0.05 |
| Overall | 53 | 254.06±88.29 | (229.72, 278.39) | 213.21±83.24 | (190.26, 236.15) | 109.64±47.59 | (96.52, 122.76) | <0.05 | <0.05 | <0.05 |

Results are stratified by periodontal disease stage, with mean, standard deviation (SD), and 95% confidence intervals (CI).

P-values were calculated using independent two-sample t-tests to evaluate group differences.
